# Supplementary material for: Foraging behaviour of an egg parasitoid exploiting plant volatiles induced by pentatomids: the role of adaxial and abaxial leaf surfaces
Source: PeerJ. 2017 May 17;5:e3326. doi: 10.7717/peerj.3326 (PMC5437855; doi:10.7717/peerj.3326)
Supplement: Data Set S1 [file peerj-05-3326-s001.pdf]

FIGURE 1a								
**set_T0**	**cnt_T0**	**test_T0**	**set_T24**	**cnt_T24**	**test_T24**	**set_T48**	**cnt_T48**	**test_T48**
1	109	155.399996	1	370.68	187.36	1	218.12	207.28
1	121.04	328.839999	1	529.12	89.84	1	45	495.56
1	116.52	128.56	1	260.52	247.52	1	208.44	31.52
1	118.28	214.720001	1	168.68	395.56	1	124.32	458.64
1	137.08	101.359999	1	500.04	51.12	1	269.48	182.8
1	174.88	219.200001	1	6.720001	27.72	1	337.56	114.36
1	93.72	395.320005	1	515.2	3.12	1	253.32	130.48
1	264.4	223.720001	1	151.84	379.12	1	246.72	237.88
1	71.64	332.520002	1	402.68	84.28	1	244.32	181.4
1	558.8	0	1	469.56	49.6	1	193.88	301.04
2	161.48	31.040001	2	418.6	88	2	584.96	1.04
2	3.519999	195.320002	2	460	117.4	2	195.32	338.84
2	551.28	33.24	2	343.72	77.36	2	134.56	442.92
2	248.16	191.960002	2	527.16	155.28	2	169.96	403.12
2	438.8	79.640002	2	249.92	104.32	2	2.52	593.28
2	156.4	231.760001	2	338.6	208.4	2	169.16	385.4
2	85.08	199.48	2	427.68	155.04	2	37.6	546.64
2	198.92	246.399998	2	356.28	164.28	2	465.28	68.24
2	165.04	420.439996	2	214.4	59.88	2	212.48	257.48
2	45.68	21.72	2	219.64	211.04	2	289.16	300.72
3	435.44	64.399998	3	363.36	145.68	3	226.08	322.76
3	343.12	191.439997	3	525.64	0.679999	3	159.84	405.92
3	82.96	659.16	3	361.68	129.64	3	226.4	225.52
3	206.04	295.199998	3	287.48	236.32	3	574.64	0
3	336.4	97.199999	3	22.76	428.36	3	246.04	206.28
3	12.52	523.280001	3	405	91.32	3	276.8	307.84
3	237.76	325.760005	3	304.92	350.28	3	537.96	0.640001
3	181.6	272.000005	3	344.76	87.72	3	413.4	30.32
3	329.92	155.920004	3	296.28	224.84	3	252.92	311.96
3	191.2	329.240001	3	169.16	260.88	3	223.88	326.96
4	262.32	156.200004	3	408.24	68.16	4	152.04	55.24
4	187.56	356.199998	4	347.8	130.48	4	231.72	346.68
4	204.28	313.16	4	563.08	32.96	4	272.72	392.36
4	137.52	191.040001	4	582.48	0	4	342.72	145.08
4	161.96	86.799999	4	172.08	43.4	4	3.84	0
4	91.92	370.999997	4	576.48	14	4	270.24	204.76
4	105.04	114.279999	4	100.56	468.68	4	22.12	526.48
4	150.52	376.520002	4	550.6	32.8	4	57.8	413.84
4	339.36	154.480001	4	3.479999	193.2	4	417.8	58.8
			4	253.52	91.28	4	101.16	407.28
			4	397.4	43.84	4	500.08	32.52

FIGURE 1b						
**set _adaxia**	**cnt _adaxi**	**test _adax**	**set _overtu**	**cnt _overtu**	**test _overturned**	
1	250.36	285.76	1	524.48	49.04	
1	263.96	223.4	1	588.52	0	
1	130.2	287.8	1	32.68	470.56	
1	351.96	170	1	340.56	181	
1	237.24	237.64	1	483.64	64.16	
1	356.6	194.84	1	349.12	95.80001	
1	272.68	280.64	1	295.48	239.4	
1	8.120001	432.88	1	353.08	146.44	
1	418.84	113.28	1	326.28	256	
1	219.84	349.28	1	158.36	97.8	
2	236.6	301.88	2	216.84	220.88	
2	312.24	213.16	2	519.96	65.32	
2	135.64	127.96	2	485.32	36.6	
2	330.32	236.12	2	184.8	408.24	
2	149.4	318.04	2	162.8	420.96	
2	157.16	282.16	2	20.72	540.04	
2	424.28	17.6	2	176.48	346.96	
2	302.52	115.92	2	271	244.04	
2	288.76	274.44	2	462.36	49.72	
2	193.88	239.28	2	359.76	217	
3	237.16	329.12	4	445.4	61.44	
3	356.44	195.2	4	40.68	403.68	
3	331.4	208.6	4	485.76	50	
3	392.68	84	4	270	7.360001	
3	364.68	693.84	4	425.76	164.72	
3	320.16	224.68	4	330.92	150.12	
3	106	470.16	4	367.24	113.12	
3	107.4	302.08	4	261.72	130.12	
3	203.92	360.28	4	206.96	354.44	
3	219.84	364.76	4	290.88	291.8	
4	231.88	199.48	5	77.72	434.68	
4	324.96	234.76	5	475.24	0	
4	268.24	74.4	5	506.16	4	
4	265.36	0	5	107.92	415.84	
4	324.4	259.88	5	522.2	43.64	
4	214.56	300.32	5	358.4	234.48	
4	194.6	381.4	5	359.76	202.36	
4	326.04	179	5	330.52	228.2	
4	262.28	258.84	5	446.8	133.64	
4	388.04	158.52	5	357.64	189.16	

FIGURE 1c

set_Murga	cnt_Murga	test_Murg	set_Nezara	cnt_Nezara	test_Nezara male
1	62.96	519.76	1	550.04	5.88
1	393.84	184.36	1	505.44	87.52
1	61.48	234.6	1	574.6	0
1	247.2	300.84	1	296.88	193.28
1	531.44	32.56	1	299.64	237.92
1	574.12	1.440001	1	337.08	79.2
1	0.28	377.8	1	3.240001	573.32
1	311.36	246.12	1	559.16	86.68
1	88.8	506.68	1	12.6	671.72
1	130.08	212	1	35.28001	528.28
2	237.92	319.04	2	372	167.24
2	153.16	75.8	2	220.2	242.96
2	30.4	388.92	2	287.4	289.8
2	276.52	176.64	2	137.4	425.08
2	422	149.28	2	179.4	361.88
2	51.6	463.92	2	163.52	421.76
2	298.92	284.56	2	252.96	337.16
2	119.36	455.36	2	391	174.04
2	4.999999	409.32	2	22.16001	529
3	44.88	497.64	2	162.4	416.76
3	140.56	403	3	218.52	256.68
3	126.92	406	3	255.8	288.16
3	58.56	498.72	3	270	282.52
3	360.2	122.16	3	116.6	355.84
3	334.4	778.68	3	0	582.88
3	47.44	543.44	3	369.44	31.88
3	430.8	147.96	3	170.56	348.92
3	482.44	102.44	4	63.12	529.56
3	51.4	481.84	4	422.76	57.48
3	281.84	116.72	4	427.96	4.959999
4	347.52	245.96	4	59.52	485.88
4	291.32	263.2	4	396.56	42.08
4	289.36	166.24	4	116.08	225.68
4	445.64	84.28	4	169.4	191.6
4	338.6	247.12			
4	17.32	559.76			
4	286.96	293.44			
4	257.72	275.52			
4	322.96	267.56			

FIGURE 2

set_FO ada	cnt_FO ada	test_FO ad	set_F adax	cnt_F adax	test_F ada	set_FO aba	cnt_FO aba	test_FO ab	set_F abax	cnt_F abax	test_F abaxial
1	320.12	7.32	1	0	452.28	1	313.28	145.68	1	183.96	278.48
1	289.92	116.24	1	400.64	185.24	1	543.04	49.48	1	110.32	465.32
1	542.52	26.6	1	292.28	76.44	1	286.72	283.08	1	54.84	299.56
1	451.52	64.92	1	265.92	301.84	1	0	586.04	1	0	353.76
1	133.52	16.4	1	103.8	473.6	1	246.88	331.08	1	215.64	256.96
1	123.8	87.52	1	219.64	242.72	1	311.68	277.52	1	182.72	316.76
1	149.64	264.72	1	198.8	377.56	1	488.44	97.64	1	261.2	299.8
1	362.2	144.72	1	195.36	142.28	2	123.48	354.56	1	454.68	43.24
1	38.56	327.04	1	496.28	97.56	2	3.56	368.76	1	420.48	44.6
1	8.76	448.8	1	439.44	119.2	2	112.56	599.64	1	233.6	162.4
2	407.72	191.36	2	522.24	1.72	2	115.52	400.4	2	263.72	296.2
2	480.56	108.96	2	587.48	3.280001	2	260.52	94.48	2	298	272.84
2	537.32	54.68	2	234.04	217.16	2	0	306.68	2	360.56	201.32
2	312.68	187.12	2	529.84	10.96	2	119.04	395.2	2	196.48	311.72
2	1.040002	567.44	2	400.32	105.4	2	529.2	0	2	256.52	231.4
2	519.4	34.72	2	408.56	142.04	2	118.04	448.16	2	327.08	202.88
2	181.52	361.28	2	348.4	232.96	2	157	50.8	2	198.64	104.28
2	450.12	82.56	2	22.16	456.84	3	0	530.8	2	439.96	91.24
2	64.92	505.84	3	5.44	1	3	21.88	512.56	2	280.96	157.24
2	245.28	130.64	3	79.28	396.16	3	47.56	493.76	2	245.48	244.12
3	366.44	206.32	3	93.12	442.36	3	65.64	1022.6	3	1.439999	584.92
3	519.12	72.6	3	62.48	313.88	3	578.12	9.2	3	273.92	305.4
3	494.88	96.68	3	78.48	465.92	3	579	4.320002	3	304.52	262.24
3	263.76	250.24	3	136.84	430.84	3	440.88	0.319999	3	218.8	139.92
3	7.880001	542.36	3	67.8	469.36	3	46.76001	401.08	3	0.120001	12.96
3	519.56	65.56	3	468.32	34.36	3	216.56	305.48	3	219.76	240.6
3	551.04	25.28	3	142.52	418.28	3	75.2	409.84	3	366.72	205.44
3	286.16	289.04	4	440.08	132.56	4	251	330.52	3	318.28	206.96
3	356.72	215.2	4	8.160001	1.040001	4	253.52	318.08	3	256.08	314.52
3	381.48	163.6	4	366.88	151.24	4	381.24	177.76	4	104.36	240.76
4	248.08	289.2	4	527	64.6	4	377.24	71.2	4	183	246.92
4	86.28	247.04	4	306.32	9.960001	4	112.88	421.44	4	487.56	53.12
4	46.72	274.4	4	77.32	519.08	4	213.2	360.96	4	358.72	175.32
4	210.16	380.8	4	193.72	161.28	4	253.04	339.28	4	89.44	417.96
4	97.84	309.88				4	13.2	572.08	4	324.92	166.8
4	154.12	317.16				6	118.44	350.68	4	119.2	466.12
4	201.72	343.92				6	10.04	462.76	5	156.6	432.48
4	106.2	100.68				6	228.52	201.92	5	218.72	365
4	358.52	125.4				6	335.48	212.08	5	242.88	272.96
4	152.84	415.72				6	92.84	141.64	5	256.56	101.68
5	176.64	303.8				6	99.28	219.32	5	93.88	313.88
5	274	168.68				6	38.4	210.8	5	408.96	167.2
5	214.8	93.4				6	43.88	439.04	5	137.36	377.4
5	293.6	141.88				6	1	591.52	5	235.56	116.52
5	413.32	170.16									
5	160.24	367.48									
5	24.44	179.12									
5	209.2	364.84									
6	334.64	247.88									
6	252.28	294.64									
6	25.88	548									
6	22.12	353.28									
6	166.92	160.84									
6	188.48	332.68									
6	4.879999	175.92									
6	148.4	277.28									
6	85.48	115.96									
6	130.04	245.52									

CHEMICAL COMPOUNDS

**R.I.**	**7.93**	**8.02**	**8.32**	**9.88**	**10.8**	**10.49**	**10.98**	**11.12**	**11.23**	**11.36**	**12.16**	**12.49**	**13.051**	**13.11**	**14.13**	**14.38**	**14.51**	**14.73**	**15.83**	**15.94**	**16.45**	**17.45**	**17.94**
**treatment**																							
ctrl	0	186191	0	0	0	0	0	0	2324	1077339	28313	0	0	21798	393448	105569	43824	0	374197	163047	3642899	13759	0
ctrl	106384	161815	0	0	241384	0	29954	548654	0	640262	28313	13242	0	470947	742569	199732	0	169882	0	959703	1869388	0	0
ctrl	0	0	0	0	0	0	0	0	93775	0	28313	0	0	201962	0	0	0	0	0	0	0	0	0
ctrl	0	0	1461509	295928	0	722696	0	541699	30917	0	344502	409567	134496	0	0	0	34413	0	0	0	8240732	56998	0
ctrl	0	0	0	0	0	0	0	0	0	0	28313	0	0	0	344344	77654	37065	0	12790882	245165	223130	0	0
foot pi	617316	2510979	0	0	0	0	0	0	0	140742	0	0	0	150635	0	0	0	0	727906	0	30656465	0	0
foot pi	0	0	0	0	0	0	0	0	0	0	0	0	0	214592	0	0	0	0	1158890	0	24789378	0	0
foot pi	0	0	611546	163028	0	318952	0	249195	70383	0	142513	137270	0	355550	0	0	0	0	4261469	0	43160064	89713	0
foot pi	0	0	0	0	0	0	0	0	0	0	0	0	0	0	102971	0	0	0	722440	76841	28233338	0	0
foot pi	0	37621	0	0	0	0	0	0	0	117760	21250	0	29150	0	157003	24725	0	0	4848879	92117	29358815	0	0
foot pi	0	0	2399665	94688	236824	0	0	651293	41610	0	513754	610229	108073	336485	0	0	55615	0	10765209	0	19593282	0	0
foot ps	0	0	0	0	0	0	0	0	0	0	0	0	0	170461	56104	14341	18080	0	0	0	29154659	0	0
foot ps	0	0	0	0	0	0	0	0	0	0	0	0	0	0	418993	94572	0	0	24573630	351231	5420920	0	0
foot ps	113933	147773	1674645	60145	113716	585857	0	461972	38597	0	274454	539330	60674	0	0	0	36019	0	0	0	0	0	0
foot ps	0	0	0	0	0	0	0	0	0	0	0	0	0	0	0	0	0	0	0	0	3188542	0	0
foot ps	0	0	90805	0	0	0	143699	48439	0	784183	0	10581	0	362921	0	0	0	320566	0	0	0	52795	0
foot ps	0	0	0	0	0	0	0	0	0	0	0	0	0	536279	0	0	0	0	3186458	0	0	0	0
opi	354215	1128364	0	0	0	0	0	0	37940	170842	0	0	0	457201	0	0	0	0	0	0	3859136	0	63101
opi	160238	816665	199348	2880	329729	14017	121901	15852	0	147346	0	0	0	213478	0	0	0	49713	0	0	12761152	0	20861
opi	0	0	0	0	0	0	0	0	0	0	0	0	0	113858	0	0	0	0	15497726	0	0	0	0
opi	0	0	0	0	0	0	0	0	0	105604	0	0	0	458359	0	0	0	247529	12832075	0	28058201	36605	0
opi	0	0	0	0	0	0	0	0	11547	1457399	0	0	0	313449	69071	0	0	0	26719620	0	35467620	95935	92446
opi	0	0	0	0	0	0	1714	0	0	293423	0	0	0	458843	0	0	0	150313	9090629	0	30905265	841263	0
opi	0	0	0	0	0	0	0	0	0	0	0	0	0	0	0	0	0	0	6650971	0	35279499	0	0
ops	26832	55154	7568	0	0	0	0	0	0	94176	77001	0	0	0	0	0	0	0	39068070	0	32251014	0	10285
ops	0	404786	0	0	0	0	0	0	0	0	0	0	0	0	0	0	0	0	4212507	0	21029671	0	0
ops	0	147933	0	0	0	0	0	0	0	120610	0	0	0	0	0	0	0	0	10739471	0	18927823	0	0
ops	0	0	0	0	0	0	0	0	0	0	0	0	0	81145	0	0	0	0	0	0	27578530	0	0
ops	0	84920	0	0	0	0	0	0	0	106529	47055	0	170994	0	0	0	0	0	0	0	1290930	64089	4079
ops	36058	587952	0	0	0	0	0	0	0	628565	0	0	0	0	0	0	0	0	0	0	0	0	0
