# Supplementary figures and images for: Foraging behaviour of an egg parasitoid exploiting plant volatiles induced by pentatomids: the role of adaxial and abaxial leaf surfaces
Source: PeerJ. 2017 May 17;5:e3326. doi: 10.7717/peerj.3326 (PMC5437855; doi:10.7717/peerj.3326)

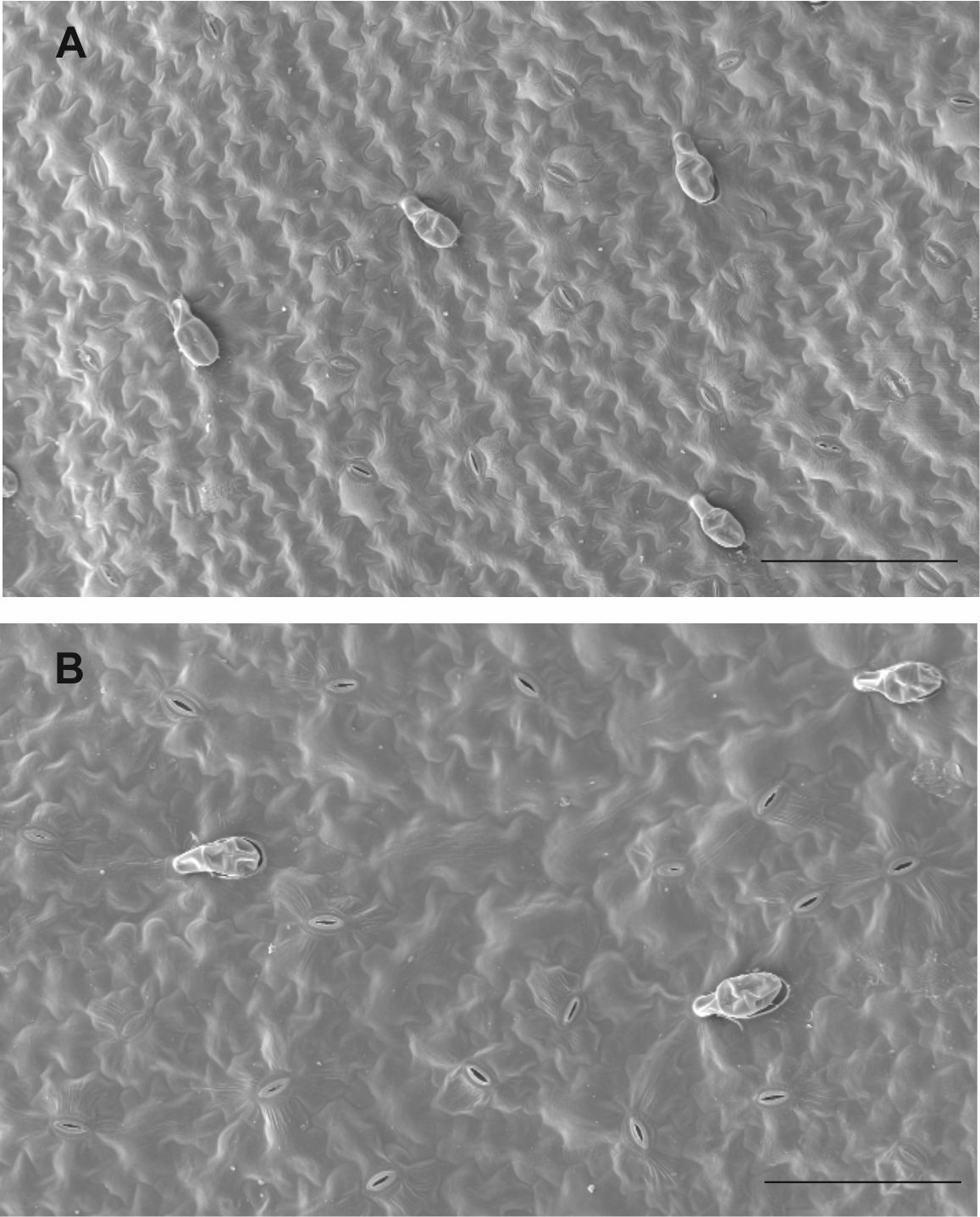

Supplement: Figure S1 — Vicia faba leaf surface. Scanning electron microscopy (SEM) of abaxial (A) and adaxial (B) leaf surface of Vicia faba plant. Bars 200 µm [file peerj-05-3326-s002.jpg]
